# Design of microaerobically inducible miniR1 plasmids

**DOI:** 10.1002/mlf2.12058

**Published:** 2023-03-20

**Authors:** Fabiola Islas, Andrea Sabido, Juan‐Carlos Sigala, Alvaro R. Lara

**Affiliations:** ^1^ Departamento de Procesos y Tecnología Universidad Autónoma Metropolitana Ciudad de Mexico México

## Abstract

Plasmid DNA manufacture is an essential step to produce gene therapy agents and next‐generation vaccines. However, little attention has been paid toward developing alternative replicons that can be coupled with large‐scale production conditions. Our results demonstrate that the miniR1 replicon can be efficiently induced by oxygen limitation when a copy of the regulatory protein RepA under control of a microaerobic promoter is used. The results are potentially attractive for industrial applications.

Plasmid DNA (pDNA) is the active pharmaceutical ingredient in the so‐called DNA vaccines^1^ and in gene therapy products. The first pDNA vaccine for use in humans, which has shown high protection against SARS‐CoV‐2, was recently approved in India^2^. Furthermore, pDNA is often used as a template for in vitro transcription to produce mRNA vaccines^3^. Therefore, current and future demands of pDNA will require efficient production processes that can be implemented on a large scale, particularly considering the physical constraints in large‐scale bioreactors, like imperfect mixing and mass transfer limitations. The oxygen required by *Escherichia coli* cells (the preferred host for pDNA production) in cultures can be difficult to meet, leading to local or global oxygen limitation. Although oxygen limitation causes undesired metabolic deviations, it can also result in increased pDNA yield (Y_pDNA/X_)^4^, ^5^. Therefore, environmental conditions in industrial bioreactors could be used to improve pDNA production. The majority of the plasmids used for DNA vaccines contain pMB1‐derived replicons, particularly pUC^1^. High pDNA yields can be obtained with pUC plasmids if the culture temperature is increased to 40–45°C, which triggers runaway replication^6^. However, this temperature increase also triggers overflow metabolism and markedly reduces the viability of cells^5^, ^7^. Moreover, the pUC origin of replication contains a cruciform sequence that is sensitive to endonuclease activity^8^, which may reduce the stability of pUC vectors. Only a few options have been proposed in addition to pUC replicons for high‐yield pDNA production. Namely, the pCOR plasmids contain the R6K replicon, and were modified to yield relatively high plasmid copy numbers (PCN) per chromosome^9^. More recently, the R1 replicon was used to construct vectors that yielded PCN of several hundreds upon thermal induction^10^. In a different report, a plasmid named pminiR1 was assembled. pminiR1 contains a synthetic R1 replicon, in which some of the natural R1 sequences were eliminated^4^. It was shown that the Y_pDNA/X_ obtained with pminiR1 was similar to that of the high PCN plasmid pUC57kan. In the present study, pminiR1 was produced in aerobic, microaerobic, and biphasic cultures with a regime change from aerobic to microaerobic conditions. pminiR1 was modified to create a microaerobically inducible version by overexpressing the positive replication control element (the protein RepA) upon oxygen limitation.

Plasmid pminiR1 contains a minimal set of sequences to allow its replication and selection (Figure 1A). For instance, the sequence of *copB*, which is a negative regulator, is also not included. Moreover, the *parB* locus originally present in plasmid R1 is not included in pminiR1. The *parB* locus contains the genes *hok* (coding for a very stable killing protein) and *sok* (coding for unstable antisense mRNA that regulates *hok* expression). In addition to antibiotic resistance, the *parB* locus provides another mechanism to stabilize plasmid R1, known as the “postsegregational killing” of plasmid free cells^11^. Therefore, the stability of pminiR1 is expected to be controlled only by antibiotic resistance.

**Figure 1:**
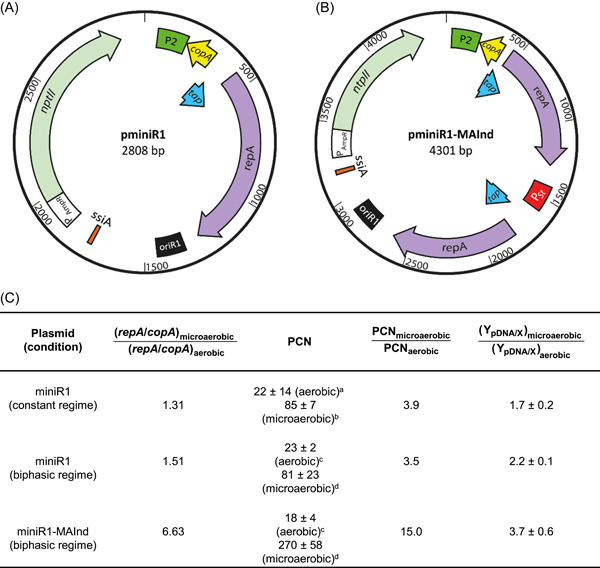
Design of the miniR1 plasmids and the main results of plasmid copy numbers and *repA* expression ratios in aerobic and microaerobic cultures. (A) Scheme of the plasmid pminiR1, containing the minimized R1 replicon. (B) Scheme of the plasmid pminiR1‐MAInd, containing an extra copy of *repA* under control of the microaerobic promoter P_*St*_. *copA*, gene coding for antisense RNA that lowers the transcription rate; *ntpII*, neomycin phosphotransferase gene; *oriR1*, origin of replication or R1 replicon; P_2_, *repA* promoter; P*_AmpR_*, promoter of the ampicillin resistance gene; P*_St_*, promoter of a globin from *Salmonella typhi*; *repA*, replication initiation protein gene; *ssiA*, single‐strand initiator (primosome assembly site); *tap*, translational activator peptide required for RepA synthesis. (C) Main results of *repA* and *copA* expression levels, Y_pDNA/X_, and plasmid copy number (PCN) per chromosome under the different conditions studied. Samples correspond to the following hours of culture: ^a^10, ^b^12, ^c^3, and ^d^11. Time profiles of the cultures are shown in Supporting Information.

Production of plasmid pminiR1 was first characterized under aerobic or microaerobic batch cultures of *E. coli* W12, which constitutively expresses the *Vitreoscilla* hemoglobin (VHb). Expression of VHb improves the growth and metabolic performance of *E. coli* under both aerobic and oxygen‐limited conditions^7^. The growth profiles of aerobic and microaerobic cultures for pminiR1 production are shown in Figure S1. Under aerobic conditions, exponential growth was observed only during the first 4–6 h. Later on, growth was approximately linear (Figure S1A). Linear growth was also reported in previous studies in the production of pDNA containing the R1 replicon^4^, ^10^. Interestingly, the pDNA yield from biomass (Y_pDNA/X_) increased markedly when the cell growth was linear (Figure S1A). The cell growth under microaerobic conditions was very slow and both pDNA concentration and Y_pDNA/X_ increased steadily during the first 10 h of culture (Figure S1B). The Y_pDNA/X_ at the end of the aerobic cultures was 6.4 ± 0.7 mg/g, while under the microaerobic regime, it reached 8.2 ± 0.4 mg/g. A previous study also reported an increase of Y_pDNA/X_ due to oxygen limitation; however, the values shown in Figure S1 are higher than those of the aforementioned report^4^. Possible causes for this are different media and temperature, as well as pH control in the present study. The final Y_pDNA/X_ in microaerobic cultures was also higher than that reported by Bower and Prather^10^, in which a plasmid containing the R1 replicon was produced in aerobic cultures at 30°C and shifted to 42°C.

To further increase Y_pDNA/X_ upon oxygen depletion, microaerobically inducible pUC replicons that substantially improve pDNA production under oxygen‐limited regimes have been designed^12^. In the present study, a similar design principle was applied: a second copy of the positive replication control gene (*repA*), placed under the transcriptional control of a microaerobic promoter (P_*St*_)^13^, was inserted in pminiR1 (Figure 1B). The microaerobically inducible plasmid was named pminiR1‐MAInd. The initial assumption was made that increased abundance of RepA would increase plasmid replication, provided that the amount of CopA does not increase in the same proportion, in agreement with previous simulations using mathematical models^14^. The *copA* promoter strength is 12.5 transcripts/min^15^, while for *repA*, it is around 1.4 transcripts/min^16^, which is nearly nine times lower. Consequently, it has been established that the synthesis of the RepA protein is a rate‐limiting factor for replication initiation^17^. Therefore, it is expected that increased *repA* expression may lead to higher PCN.

The production of pminiR1 and pminiR1‐MAInd was evaluated under biphasic conditions, as described in Materials and methods in Supporting Information. The result showed the growth profile of the biphasic culture of pminiR1 and pminiR1‐MAInd (Figure S2). The Y_pDNA/X_ increased for both plasmids after change in the regime to microaerobic conditions, reaching values higher than those achieved under constant regimes. This is advantageous for large‐scale cultures, where a gradual transition to microaerobic conditions would occur. The maximum Y_pDNA/X_ was obtained at 13 h of culture for both plasmids, reaching 9 ± 1 mg/g for pminiR1 and 12 ± 1 mg/g for pminiR1‐MAInd (Figure S2). This means increases of 33% and 84% over the original plasmid in biphasic and in fully aerobic cultures, respectively.

To confirm the relation between the increased Y_pDNA/X_ and *repA* expression, the PCN and the ratio of *repA* copies to *copA* copies per cell were measured and are reported in Figure 1C. In cultures at constant regimes, the *repA*/*copA* expression ratio increased 31% under the microaerobic condition, compared to the aerobic condition, which led to a 3.9‐fold increase of PCN. The absolute PCNs are lower than those reported by Bower and Prather^10^. However, those authors performed cultures at 30°C, which improves the stability of low‐copy‐number plasmids in *E. coli*. Microaerobic conditions were also used for the production of the pUC plasmid (pVAX1) in batch mode. The authors reported a 61% increase of Y_pDNA/X_, compared to aerobic conditions^7^, which is similar to the results shown in Figure 1C. Microaerobic cultures at a very low oxygen transfer rate (OTR, 10 mmol/l h) resulted in five times higher Y_pDNA/X_ values for pVAX1, compared to aerobic cultures at OTR_max_ of 110 mmol/l h^18^. Therefore, it may be possible to improve the results shown in Figure 1C by decreasing the OTR_max_ of the culture.

The biphasic regime resulted in increased *repA*/*copA* expression ratios, compared to constant regime cultures. Notwithstanding the increase of Y_pDNA/X_ of pminiR1 observed in biphasic cultures compared to constant regimes, the PCN increase was almost unchanged (Figure 1C), meaning that there was no direct equivalence between PCN and Y_pDNA/X_. This was also observed by Bower and Prather^10^ and contrasts with pUC plasmids^12^. The *repA*/*copA* expression ratios in cells bearing pminiR1‐MAInd were more than 4‐fold higher than that for pminiR1, in agreement with substantial increases of PCN and Y_pDNA/X_ (Figure 1C). The PCN values obtained by the end of biphasic cultures of cells bearing pminiR1‐MAInd under microaerobic condition were 3‐ and 12‐fold higher than those attained in a constant microaerobic and aerobic regime, respectively, using pminiR1. This change is substantially higher than that reported for a microaerobically inducible pUC plasmid. In this report, the PCN was 2.3‐fold higher during the microaerobic phase, compared to the aerobic phase of a fed‐batch culture^12^. This could be related to the fact that the positive control molecule for miniR1 is a protein, contrary to the case of pUC plasmids, in which the PCN is controlled by RNA. The half‐life of the majority of RNA molecules in *E. coli* is between 3 and 8 min^19^, while the half‐life for proteins (excluding abnormal or unstable proteins) is several hours^20^. Therefore, the additional expression of RepA under microaerobic conditions could lead to more stable effects than the expression of RNA.

Plasmid topology is a relevant factor when pDNA is used as an active pharmaceutical ingredient^1^. It is generally recommended that at least 80% of the pDNA is present in the supercoiled isoform (SCF)^1^. We analyzed the supercoiled content of both plasmids obtained from the different regimes. The result showed that under both aerobic and microaerobic regimes, the sc‐pDNA fraction of pminiR1 was 80%. In biphasic cultures, the sc‐pDNA fraction during the aerobic phase of the sc‐pDNA was 80%, while in the microaerobic phase, it increased to 90%. Similar results were obtained for pminiR1‐MAInd (Figure S3).

Here, we present an alternative to deal with microaerobic conditions that can easily occur during the production of pDNA at any culture scale, from shake flasks to large bioreactors. The R1 replicon can be an alternative to traditional pUC replicons. Therefore, a minimal R1 plasmid was modified to induce replication upon transition to microaerobic conditions. In the experiments presented here, it is considered that microaerobic conditions are present when dissolved oxygen tension (DOT) is below 10%. The modified miniR1 contains an extra copy of the gene *repA* under control of the microaerobic promoter P_*St*_. Although the DOT for optimal induction of P_*St*_ has not been reported, it has been demonstrated that the promoter from the *Vitreoscilla* hemoglobin is maximally induced at DOT below 5%^21^. Since both promoters control the expression of microbial globins, we chose DOT of 2% for induction of *repA* as an initial approximation. While precise control at such a low DOT in industrial bioreactors is difficult, our experiments show the differences between two contrasting conditions: fully aerobic and microaerobic conditions.

The presented results demonstrate that the transition to microaerobic conditions is better than constant microaerobicity to increase the production of plasmids containing the minimal R1 replicon. Furthermore, our biodesign proved that overexpression of the gene *repA* is an efficient way to increase pDNA yields and PCN upon transition to oxygen limitation. Calculated over the entire whole culture time of cultures shown in Supporting Information, the global productivity in biphasic cultures was 1.61 ± 0.12 mg/l h for pminiR1 and 1.71 ± 0.15 for pminiR1‐MAInd, which is an increase of only 6.2%. However, the SCF increased from ~80% to ~90%. Therefore, the increased pDNA yields and SCF of pminiR1‐MAInd make it advantageous for downstream operations. Overall, we show that the minimized R1 replicon can be an interesting option to the traditional pUC replicon for achieving high yields. Microaerobic conditions can increase the PCN of pminiR1. The designed inducible plasmid is therefore an alternative for oxygen transfer limitations in bioreactors, and therefore, can help to efficiently scale up pDNA production processes.

## AUTHOR CONTRIBUTIONS

All authors contributed to the study conception and design. Material preparation, data collection, and analysis were performed by Fabiola Islas. RT‐qPCR analyses were performed by Fabiola Islas and Andrea Sabido, and supervised by Juan‐Carlos Sigala. The first draft of the manuscript was written by Fabiola Islas and Alvaro R. Lara, and all authors commented on previous versions of the manuscript. All authors read and approved the final manuscript.

## ETHICS STATEMENT

This article does not contain any studies with human participants or animals performed by any of the authors.

## CONFLICT OF INTERESTS

The authors declare no conflict of interests.

## Supporting information

Supplementary information.

## Data Availability

The data that support the findings of this study are available from the corresponding author upon reasonable request.
