# Supplementary material for: Design of microaerobically inducible miniR1 plasmids
Source: mLife. 2023 Mar 20;2(1):101–4. doi: 10.1002/mlf2.12058 (PMC10989972; doi:10.1002/mlf2.12058)
Supplement: Supplementary file 1 — Supplementary information. [file MLF2-2-101-s001.docx]

**Supplementary file to:**

**Design of microaerobically inducible miniR1 plasmids**

Fabiola Islas, Andrea Sabido, Juan-Carlos Sigala, Alvaro R. Lara^*^

**Materials and methods**

**Host strain.** *Escherichia coli* W3110*recA*^-^ *vgb*^+^, a derivative of *E. coli* W3110 was used as production host. In this strain, the gene *recA* was deleted and the *vgb* gene coding for the *Vitreoscilla stercoraria* hemoglobin (VHb) was chromosomally integrated and placed under transcriptional control of the promoter P*_trc_*. For simplicity, the strain is denominated here as W12. The strain was transformed by electroporation with the plasmids described below and conserved in 40 % glycerol at -80 °C.

**Plasmids.** pminiR1 (2808 bp) contains the minimal elements of the R1 replicon for replication control, like the gene coding for the replication initiation protein (RepA) and *copA*, which codes for antisense RNA that binds to its target in the leader region of the RepA mRNA, lowering the replication rate. pminiR1 lacks the gene *copB*, which is a negative replication control element in plasmid R1. pminiR1 also contains a kanamycin-resistance marker. Details on the design and construction of pminiR1 are provided elsewhere^4^. pminiR1-MAInd (4301 bp) is based on pminiR1 and includes an extra copy of *repA* under control of the microaerobic promoter P*_St_*. Such promoter controls the expression of a globin in *Salmonella typhi*^13^, and displays higher activity upon oxygen limitation than other endogenous and heterologous promoters in *E. coli*^13^. The construction was purchased from GenScript (NJ, USA).

**Culture medium and bioreactor conditions.** All cultures were performed in lysogeny broth 2X (LB, Sigma, MO, USA) supplemented with 50 mg/L kanamycin sulphate (Sigma, MO, USA). Precultures were carried out by inoculating 100 μL of cryopreserved cells in 50 mL of LB medium contained in 500 mL shake flasks and grown overnight at 37 °C, 250 rpm. Bioreactor cultures were carried out in a 1 L Biostat A Plus stirred tank bioreactor (Sartorius BBI, Melsungen, Germany) with a working volume of 0.5 L, at 37 °C, pH 7.0 (controlled by automatic addition of 15% NH_4_OH and 10% H_3_PO_4_). The initial cell density was set to OD_600nm_ of approx. 0.8 units. Air was sparged at 0.5 L/min through a stainless steel microsparger (Sartorius BBI, Melsugen, Germany). Dissolved oxygen tension (DOT) was measured with a polarographic sensor (Hamilton, Reno, NV, USA). The sensor was thoroughly cleaned with deionized water, membrane integrity visually checked, and refilled with fresh electrolyte before each culture. Previous to inoculation, the sensor was calibrated at 0 % (after sparging pure nitrogen at 0.5 L/min for at least 20 min) and 100 % saturation (after sparging pure air at 0.5 L/min for at least 20 min). Then, 0 % air sat. was checked by sparging nitrogen again. In biphasic cultures, DOT was maintained above 30% air saturation during the first 4 h (aerobic phase) and then shifted restricted oxygen supply (DOT ≤ 2%, microaerobic phase). DOT was controlled by a PI controller in the agitation cascade mode (*t_i_* = 50 s; *x_p_* = 140%; *t_D_* = 0 s; dead band = 0.1%) using the MFCS/DA software (Sartorius BBI, Melsungen, Germany). Cell growth was followed by measuring OD_600nm_ in a BioPhotometer Plus (Eppendorf, Wesseling-Berzdorf, Germany). Biomass concentration was determined as the dry cell weight. All cultures were performed in duplicate.

**pDNA analyses.** Samples for pDNA were centrifuged for 3 min at 7000 x g, and the supernatant was discarded. Cell pellets were stored at -20 °C for no more than two weeks. pDNA was isolated using a QIAgen Spin Miniprep kit (Qiagen, Hilden, Germany) following the manufacturer’s instructions and eluted with 70 μL of EB buffer at 70 °C to enhance pDNA recovery. Total double-stranded DNA was measured spectrophotometrically using a NanoDrop 2000 (Thermo Scientific, MA, USA). pDNA supercoiled fraction (sc-pDNA) was determined by electrophoresis of 100 ng of pDNA in 1% agarose gel in TAE buffer for 1 h at 80 V. sc-pDNA was identified by the bands that migrate faster than the linear DNA reference and correspond to the covalently closed circular (ccc) monomer. Image analyses were performed with the Image J software (NIH, Bethesda, MD, USA). The sc-pDNA fraction was calculated as the ratio of the product of area and colour intensity of such band to the product of area and colour intensity of the lane in which the sample was loaded.

**Total DNA purification.** Samples for total DNA isolation were centrifuged for 3 min at 7000 x g, and the supernatant was discarded. Cell pellets were stored at -20 °C. Total DNA was isolated using a QIAamp DNA mini kit (Qiagen, Hilden, Germany) following the manufacturer’s instructions. Total DNA was measured spectrophotometrically using a NanoDrop 2000 (Thermo Scientific, MA, USA). The total DNA sample concentration was then normalized to 2 ng/μL.

**Plasmid Copy Number (PCN) determination.** The PCN was determined through quantitative reverse transcription polymerase chain reaction (RT‐qPCR) absolute quantification. Standard curve experiments were executed in a 7500 Real‐Time PCR System (Applied Biosystems, CA, USA) using SYBR Green (Thermo Scientific, MA, USA). PCN was determined using an 82 nucleotides amplicon of the *kan* gene generated from primers kan-F (5´ CATTCGTGATTGCGCCTGAG 3´) and kan-R (5´ CCGGTTGCATTCGATTCCTG 3´). The copies of a chromosomal gene were quantified through a 101-nucleotide amplicon of the *ihfB* gene generated from primers ihfB-F (3´ GCCAAGACGGTTGAAGATGC 5´) and ihfB-R (5´GAGAAACTGCCGAAACCGC 3´). The cycle used for amplification of *kan* and *ihfB* consisted of a precycling step (10 min at 95 °C) and 40 cycles of denaturation (15 s at 95 °C) and amplification (60 s at 62 °C). After amplification was completed, a melt curve step from 60 °C to 95 °C was executed at a rate of 0.1 °C/s. Reactions were set up at 12 μL as follows: 6 μL SYBR Green PCR Master Mix reagent (Thermo Scientific, MA, USA); 1 μL of 200 nM primers; 4 μL milli-Q water and 1 μL of the sample. For each qPCR run were used six 10‐fold serial dilutions of a 0.02 ng/μL standard of the purified amplicons and a no template control (NTC). Standards, NTCs and samples were assayed in triplicate.

**RNA isolation, purification, and quality control.** RNA was isolated using the phenol-chloroform method. RNA was measured spectrophotometrically using a NanoDrop 2000 (Thermo Scientific, MA, USA) and subjected to DNase treatment using the Turbo DNA free kit (Thermo Scientific, MA, USA) following the recommendations of the manufacturer. Samples were verified for chromosomal DNA contamination by not amplifying *bla* gen by PCR. The samples were separated by electrophoresis in a 0.8 % agarose gel stained with SYBR Green Safe (Invitrogen, CA) at 80 V for 1 h. None of the control samples presented bands after UV light exposure in a Gel Logic 200 Imaging System (Kodak). RNA integrity was evaluated by chip microfluidics electrophoresis in a 2100 Bioanalyzer (Agilent, CA, USA) using the RNA 6000 Nano Reagents Part I and the RNA Nano Chips. The RNA integrity number (RIN), was automatically calculated by the 2100 Expert Software (Agilent, CA, USA). All the samples had RINs between 7 and 9.

***repA* and *copA* expression levels.** The expression levels of *repA* and *copA* were determined by RT‐qPCR absolute quantification in a 7500 Real‐Time PCR System (Applied Biosystems) using SYBR Green. cDNA samples were diluted threefold with milli-Q water to improve liquid handling and to minimize inhibitor concentrations. For *repA* determination, oligonucleotides repA-F (5´ TCCGGTGCAGGAAAACTCTC 3´) and repA-R (5´ AGCGGGTCATATTCCGTCTG 3´) were used to generate a 96 nucleotides amplicon. The cycle used for amplification consisted of a precycling step (10 min at 95 °C) and 40 cycles of denaturation (15 s at 95 °C) and amplification (60 s at 55 °C). After amplification was completed, a melt curve step from 60 °C to 95 °C was executed at a rate of 0.1 °C/s. For *copA* determination, oligonucleotides copA-F (5´ AAAGCAAAAACCCCGATA 3´) and copA-R (5´ ATAGCTGAATTGTTGGCT 3´) were used to generate a 92 nucleotides amplicon. The cycle used for amplification consisted of a precycling step (10 min at 95 °C) and 40 cycles of denaturation (15 s at 95 °C) and amplification (60 s at 46 °C). After amplification was completed, a melt curve step from 60 °C to 95 °C was executed at a rate of 0.1°C/s. The total volume of each reaction was 12 μL with the following composition: 6 μL SYBR Green PCR Master Mix Reagent (Thermo Scientific, MA; USA); 1 μL of 200 nM primers; 4 μL milli-Q water; and 1 μL of sample. For each qPCR run, six dilutions of a standard of the purified amplicons plus a NTC were included. Standards, NTCs and samples were assayed in duplicate.

**cDNA synthesis.** cDNA from *repA* and *copA* was generated with the RevertAid MinusFirst Strand cDNA Synthesis Kit (Thermo Scientific, MA, USA) using primers repA-R and copA-R complementary to the 3′ ends of the genes. The final primer concentrations were 10 μM in the reaction mixture, and the RNA sample concentration was normalized to 75 ng/μL. The synthesis was carried out at 42 °C for 65 min, followed by a termination step at 70 °C for 10 min in an Arktik 5020 Thermal Cycler (Thermo Scientific, MA, USA).

**RT‐qPCR calculations.** The standard copies, copy number and amplification efficiencies were calculated as described earlier^12^. The PCN was calculated as the ratio of *kan* copies per cell and *ihfB* copies per cell. Similarly, the *repA*/*copA* ratio was determined by dividing *repA* copies per cell by *copA* copies per cell.

**Supplementary Figures**


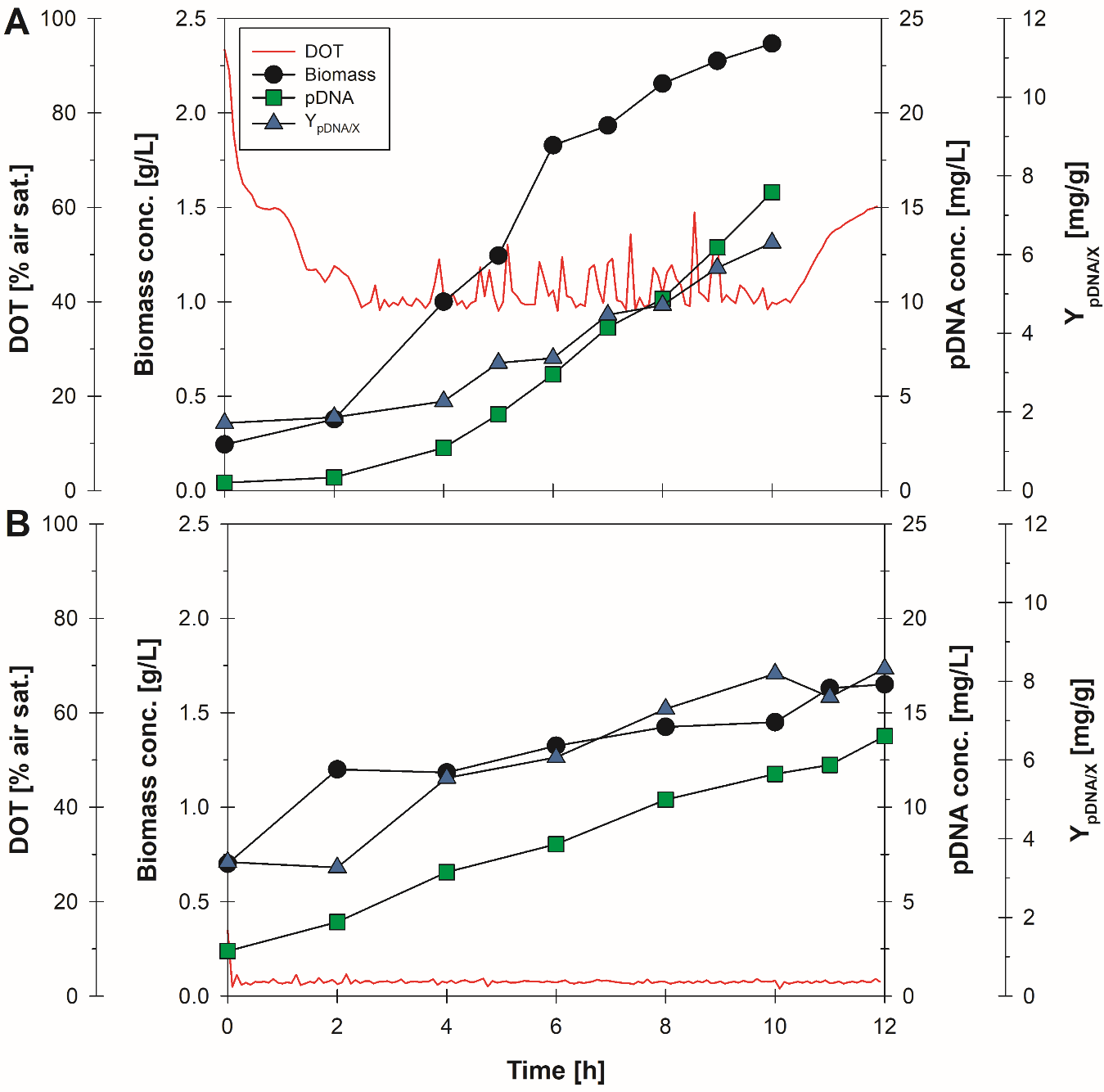


**Figure S1.** Growth profiles, pDNA production, pDNA yield from biomass (Y_pDNA/X_), and DOT in cultures of W12 pminiR1 under constant regimes. **A**: Aerobic conditions. **B**: Microaerobic conditions. For clarity, results from only one culture are shown.


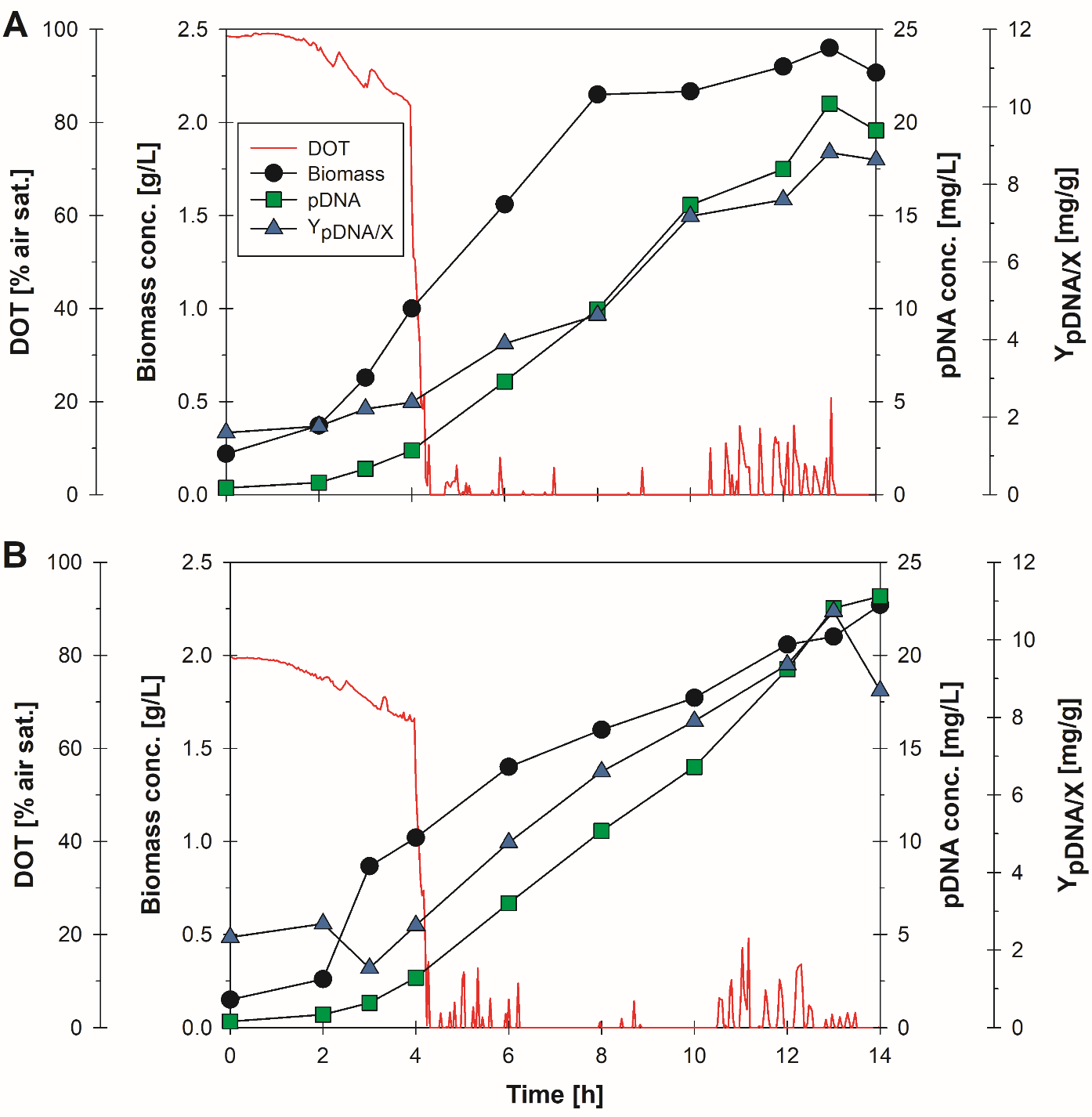


**Figure S2.** Growth profiles, plasmid production, yield on biomass (Y_pDNA/X_), and DOT under biphasic regimes by strain W12 at 37°C in biorreactor. **A**: pminiR1. **B**: pminiR1-MAInd. For clarity, results from only one culture are shown.


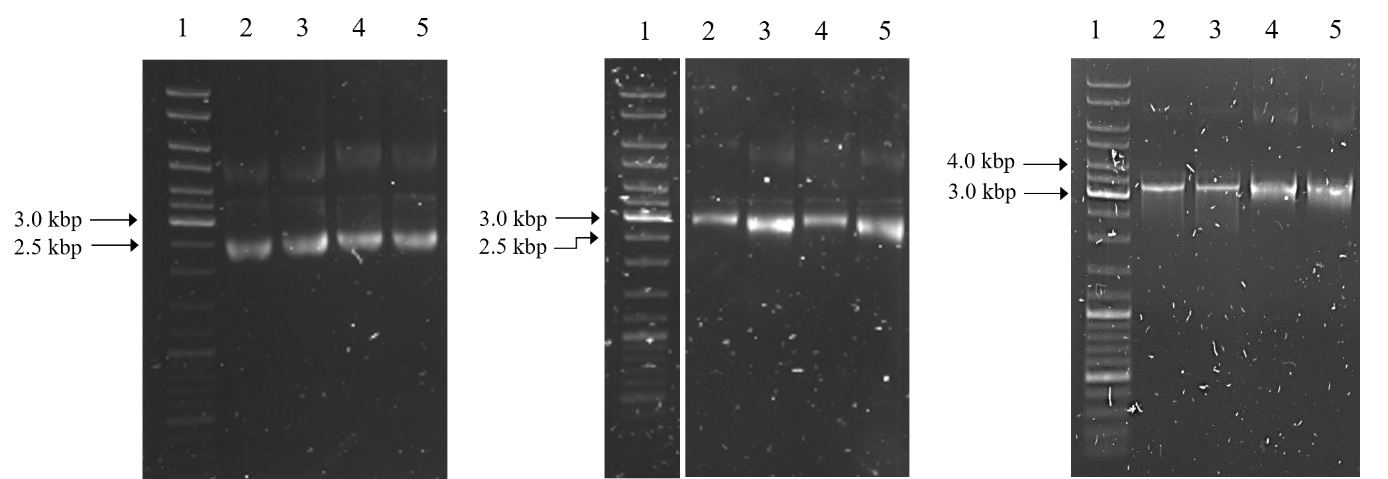


**Figure S3.** Agarose gels for sc-pDNA analyses**. Left.** Analysis of pminiR1 in cultures at constant regimes. Lane 1: GeneRuler DNA Ladder Mix (Sigma Aldrich); lanes 2 and 3, aerobic cultures (duplicates); lanes 4 and 5, microaerobic cultures (duplicates). **Center.** Analysis of pminiR1 in cultures with biphasic regimes. Lane 1: GeneRuler DNA Ladder Mix (Sigma Aldrich); lanes 2 and 4: aerobic phase; lanes 3 and 5: microaerobic phase. **Right.** Analysis of pminiR1-MAInd in cultures with regimes. Lane 1: GeneRuler DNA Ladder Mix (Sigma Aldrich); lanes 2 and 3: aerobic phase; lanes 4 and 5: microaerobic phase. Sampling times correspond to that of Figure 1C.
